# Integrated 64 pixel UV image sensor and readout in a silicon carbide CMOS technology

**DOI:** 10.1038/s41378-022-00446-3

**Published:** 2022-10-25

**Authors:** Joost Romijn, Sten Vollebregt, Luke M. Middelburg, Brahim El Mansouri, Henk W. van Zeijl, Alexander May, Tobias Erlbacher, Johan Leijtens, Guoqi Zhang, Pasqualina M. Sarro

**Affiliations:** 1 Laboratory of Electronic Components, Technology and Materials (ECTM), Department of Microelectronics, Delft University of Technology, Delft, The Netherlands; 2 Fraunhofer Institute for Integrated Systems and Devices Technology IISB, Erlangen, Germany; 3 Lens R&D, Noordwijk, The Netherlands

**Keywords:** Optical sensors, Electrical and electronic engineering

## Abstract

This work demonstrates the first on-chip UV optoelectronic integration in 4H-SiC CMOS, which includes an image sensor with 64 active pixels and a total of 1263 transistors on a 100 mm^2^ chip. The reported image sensor offers serial digital, analog, and 2-bit ADC outputs and operates at 0.39 Hz with a maximum power consumption of 60 μW, which are significant improvements over previous reports. UV optoelectronics have applications in flame detection, satellites, astronomy, UV photography, and healthcare. The complexity of this optoelectronic system paves the way for new applications such harsh environment microcontrollers.

## Graphical abstract

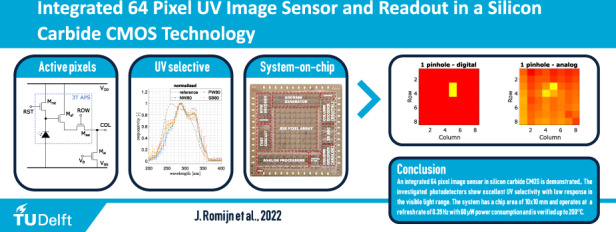

## Introduction

**Fig. 1:**
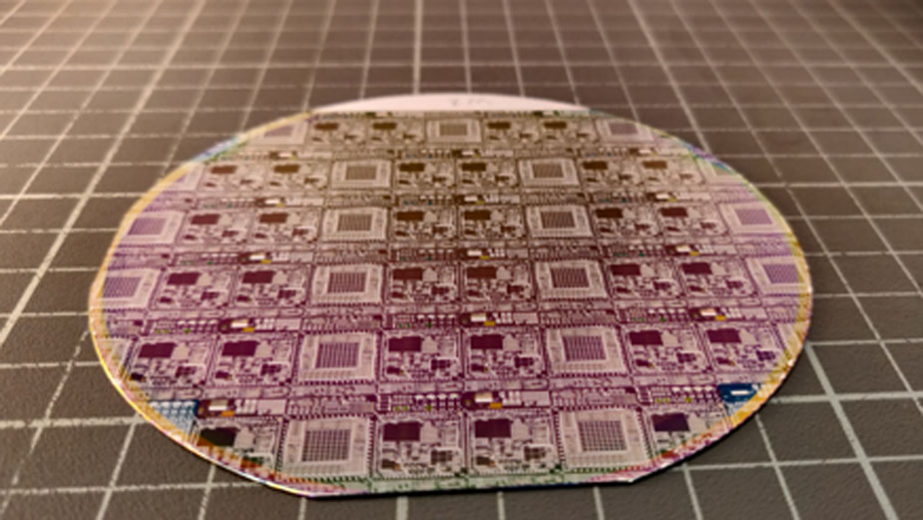
Silicon carbide device wafer. Photograph of the multi-project four-inch silicon carbide device wafer that was fabricated in the Fraunhofer IISB 6 μm 4H-SiC CMOS technology.

The ultraviolet (UV) spectrum is divided into the UV-A (315–400 nm), UV-B (280–315 nm), and UV-C (200–280 nm) sections. Reliable UV photodetection without distortion by other spectral bands is challenging when using conventional silicon-based technologies, as its sensitive spectral bandwidth is wide. Photodetectors in silicon for UV detection exist^1^, but are also sensitive to visible light. Alternatively, implementations based on silicon-on-insulator^2^ or ultrashallow junctions^3^ exist, which exploit the thin film that transmits light with longer wavelengths. Furthermore, UV selectivity can be achieved by elegant readout approaches, such as the use of a differential method^4,5^. By instead using wide-bandgap materials as the substrate, the sensitive spectral bandwidth of the semiconductor is reduced to shorter wavelengths. This furthermore comes with the potential of applications in deeper UV or in higher temperature environments. Here, we report the results of an image sensor with readout electronics in silicon carbide (Fig. 1).

A selection of wide-bandgap materials and their properties is listed in Table 1. Note that the excitation wavelength is inversely proportional with the bandgap of the material and a detector that is selectively sensitive to deep UV should therefore be implemented in a material with a wide bandgap^6^. Combining realized devices in these substrates with on-chip circuitry, ideally asks for complementary devices. An often favored circuit technology for on-chip readout is CMOS, due to its low power operation. When considering widely used CMOS technologies, the ratio between electron and hole mobility should be close to 1 as well as high absolute mobility values for fast devices. Note that there are significant differences in the ratio between electron and hole mobility, which would negatively impact active device performance and matching of complementary devices. From that point of view, diamond is the best candidate by far, followed by silicon carbide.

**Table 1:** Overview of a selection of parameters of semiconducting materials, including silicon (Si), 4H polytype of silicon carbide (4H-SiC), 6H polytype of silicon carbide (4H-SiC), zinc oxide (ZnO), galium nitride (GaN), aluminum nitride (AlN) and diamond (Dia.)^1,35–37^. A complete overview was listed by Monroy et al.^1^.

	Si	4H-SiC	6H-SiC	ZnO	GaN	AlN	Dia.
Bandgap [eV]	1.12	3.2	2.86	3.35	3.39	6.2	5.5
Excitation wavelength [nm]	1170	387	434	370	366	200	225
Melting point [°C]	1410	2557	2557	1969	2518	2752	3500
Electron mobility [cm^2^ V^−1^ s^−1^]	1400	950	400	200^a^	1000	135	2200
Hole mobility [cm^2^ V^−1^ s^−1^]	600	120	75	−^a^	30	14	1600
Electron/hole mobility ratio [-]	2.33	7.92	5.33	−^a^	33.3	9.64	1.38
Thermal conductivity [W cm^−1^ K^−1^]	1.5	4.9	4.9	0.54	1.3	3.19	20

^a^Synthesis of single-crystal samples remains challenging

**Fig. 2:**
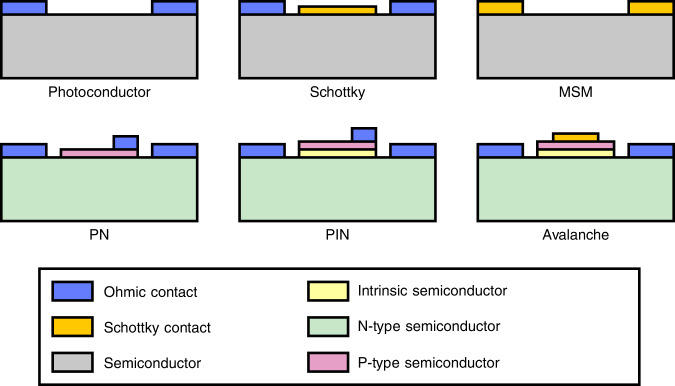
Photodetector architecture. Schematic illustrations of several different semiconductor photodetector types^1,35^. Note that the doped regions are stacked in these illustrations, but implementation in the substrate through ion implantation is also possible.

Over the past decades, silicon carbide has been a popular material in power electronics research^7,8^ as well as application in harsh environment sensing and ultraviolet detectors^9–13^. Extensive research effort was made on silicon carbide photodetectors, as they make excellent visual-blind UV detectors. Over the years the technology has come a long way, leading for example to a UV-index monitoring demonstrator board^14^ and UV imager^15^. Reliability studies on the aging effects show promising results, revealing little to no change in the device performance^16–18^. The schematic cross-section structures of the most widely used types of semiconductor photodetectors are illustrated in Fig. 2. This includes the photoconductor, Schottky, metal–semiconductor–metal, p-type/n-type doped regions (PN) junction, p-type/intrinsic/n-type doped regions junctions, and avalanche photodiode. The operating principles of these different semiconductor photodetector types are very similar as they all rely on the fundamental photoelectric effect. A selection of different photodetector implementations is provided in Table 2. The responsivity, reported in the research, is listed for specific wavelengths. The peak responsivity is close to a wavelength of 300 nm for all devices. Another promising exotic topology is the graphene-based silicon carbide Schottky photodiode, reaching extremely high responsivity values^19^. Furthermore, additions such as microlenses integrated on top of photodiodes are demonstrated^20^ to increase the external quantum efficiency and, as a result, the responsivity. None of the listed photodetectors was previously integrated with on-chip readout.

**Table 2:** A short overview of the extensive research already done on silicon carbide photodetectors. The listed responsivity values are rough estimates, taken from the reported graphs. Note that the biasing conditions are not equal for each reported work. Some works only reported quantum efficiency values or no spectral analysis.

Technology	Responsivity [A W^−1^]			Material	Researchers	Year
	250 nm	300 nm	350 nm			
MSM	−^a^	−^a^	−^a^	4H-SiC	Su et al.	2002^38^
Schottky	0.025	0.065	0.06	6H-SiC	Sciuto et al.	2017^39^
Schottky	0.019	0.046	0.006	4H-SiC	Mazzillo et al.	2016^40^
Schottky	0.018	0.044	0.01	4H-SiC	Mazzillo et al.	2014^41^
Schottky	0.018	0.042	0.01	4H-SiC	Sciuto et al.	2014^17^
Schottky	0.048	0.099	0.02	4H-SiC	Mazzillo et al.	2009^42^
Schottky	0.16	0.13	0.04	4H-SiC	Sciuto et al.	2006^43^
Schottky	−^a^	−^a^	−^a^	4H-SiC	Hu et al.	2006^44^
PN	0.10	0.10	0.015	4H-SiC	Matthus et al.	2017^26^
PN	0.07	0.1	0.06	4H-SiC	Prasai et al.	2012^18^
PN	0.13	0.12	0.05	6H-SiC	Brown et al.	1993^45^
APD	0.08	0.08	0.02	4H-SiC	Yang et al.	2017^46^
APD	0.082	0.09	0.005	4H-SiC	Liu et al.	2008^47^
APD	80	50	4	4H-SiC	Yan et al.	1999^48^
SPAD	0.3^b^	0.15^b^	0.02^b^	4H-SiC	Hu et al.	2008^49^
SPAD	0.2^b^	0.15^b^	0.03^b^	4H-SiC	Xin et al.	2005^50^

^a^No spectral analysis are reported

^b^Only quantum efficiency values are given [e-/photon]

The implementation of integrated low-voltage circuitry in silicon carbide has been feasible for over a decade, although the accessibility to the technologies has remained limited. The most promising technologies under development today are a BJT technology^21,22^ for high temperature and harsh environments, called HOTSiC, at the KTH in Sweden and a 4H-SiC CMOS^23–25^ at the Fraunhofer Institute for Integrated Systems and Devices Technology (IISB), based in Erlangen, Germany. The latter has full capability for designs with complementary devices, while the BJT technology is NPN only with pull-up resistors. Fraunhofer IISB already demonstrated UV photodetectors^26–28^, that allow integration with the CMOS fabrication technology. However, at present the only previously reported optoelectronic system in SiC, was achieved in the BJT technology at the KTH in Sweden by Hou et al.^15^. This system includes 256 addressable pixels, 1959 transistors, and runs at 8.25 W and 7.7 mHz.

This work reports on the first on-chip CMOS optoelectronic integration in silicon carbide, using UV photodetectors and CMOS readout circuitry to implement a 64 pixel image sensor in the Fraunhofer IISB 6 μm 4H-SiC technology^23^. Three photodetector implementations are reported and show excellent wafer-level yield and uniformity. One of these photodetectors implementations is used in the image sensor, which is based on previous work in a comparable silicon-based CMOS technology^29^ and previously reported silicon carbide basic CMOS circuit blocks^23^. The reported silicon carbide optoelectronic system is one of the largest device count implementations in silicon carbide to date, with 64 photodetectors and 1263 transistors, and has serial digital, analog, or 2-bit ADC output options.

## Materials and methods

The Fraunhofer IISB 6 μm 4H-SiC CMOS technology^23^ includes a double-well front-end-of-line (FEOL) implemented by ion implantation and a single-level interconnect back-end-of-line (BEOL). The FEOL layers include the n-well (NW), p-well (PW), shallow n-type (SN), and shallow p-type (SP), which are activated by a 1700 °C anneal for 30 min. The highly doped shallow layers both have doping concentrations of 5 × 10^19^ cm^−3^ with a depth of 0.3 μm. The low doped wells are much deeper and have doping concentrations of 1 × 10^16^ cm^−3^ and 1 × 10^17^ cm^−3^ for the NW and PW, respectively. The BEOL incorporates a single Ti/Al/Ti interconnect layer, but the polysilicon layer MOSFET gate material offers some more interconnect complexity. The ohmic contact to the SN and SP layers is implemented using 50 nm NiAl and 80/300 nm Ti/Al silicides respectively.

**Fig. 3:**
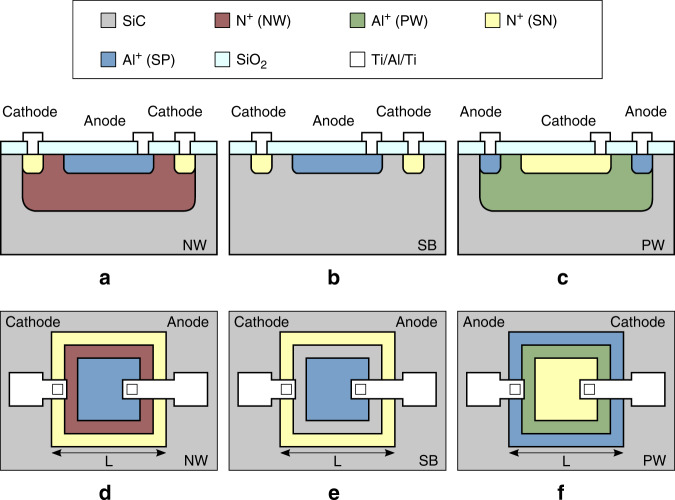
Photodiode design. Schematic illustration of three different vertical PN photodiodes in the Fraunhofer IISB technology, using the existing CMOS design layers. The implementations are inside the n-well (NW), substrate (SB), and p-well (PW), of which the **a**–**c** cross-sections are provided with the **d**–**f** top views. Note that the substrate is n-type of similar dopant concentration as the n-well.

Using the Fraunhofer IISB CMOS technology design layers, three different vertical PN photodiodes are considered. The schematic cross-section and top view of these architectures are listed in Fig. 3 and include implementations inside the NW, substrate (SB), and PW. The wells are contacted using highly doped guard rings, to achieve ohmic contacts and help reduce potential cross-talk between devices. The square photodetector dimension *L* is measured at the guard ring and is used to label the different devices.

An estimation of the generated photocurrent per unit area is used to predict the photocurrent magnitude in the design. For this purpose, a responsivity report on a 2.6 mm^2^ SiC UV photodetector in the same Fraunhofer IISB technology^26–28^ is taken as a reference. The responsivity *R*(*λ*) is multiplied by the UV light source irradiance *P*_*opt*_(*λ*) for a set of points followed by the uniform midpoint Riemann sum, resulting in

1 $${I}_{0}\approx \mathop{\sum }\limits_{i=1}^{N}{P}_{opt}\left({\lambda }_{i}^{* }\right)R\left({\lambda }_{i}^{* }\right)\Delta \lambda .$$

**Fig. 4:**
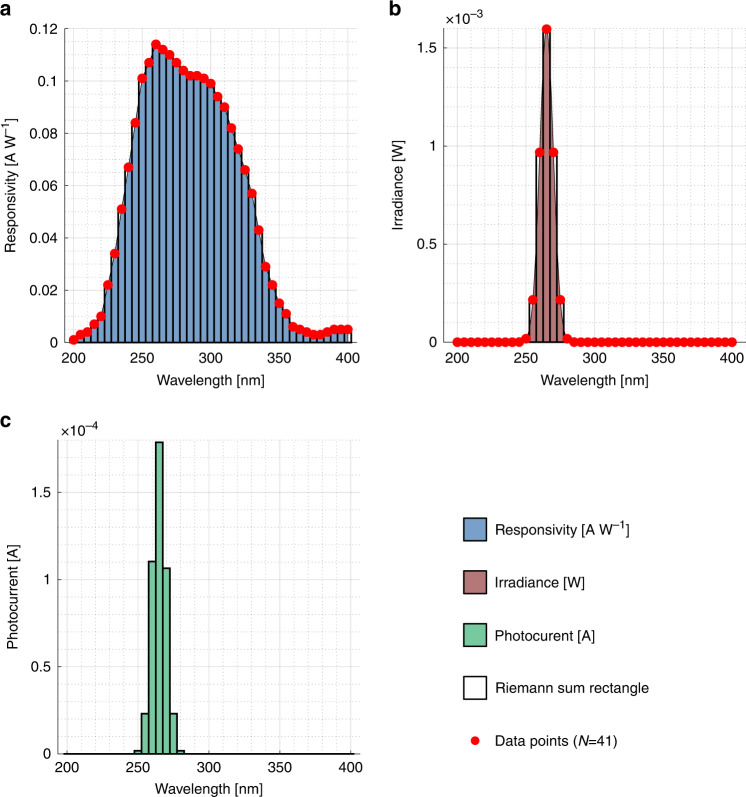
Radiometry estimation. Spectral information on the **a** Fraunhofer IISB 2.6 mm^2^ SiC UV photodiode^26–28^ responsivity *R*(*λ*), **b** the UV LED irradiance *P*_*opt*_(*λ*) of four discrete diodes with 5 mW radiant flux each and **c** the result of ${P}_{opt}\left({\lambda }^{* }\right)R\left({\lambda }^{* }\right)$ that gives the corresponding photocurrent generation.

Four discrete 5 mW SMD3535 265 nm UV-C LEDs, with a full width at half maximum of 10 nm, are used in the device measurements. The spectral information is reported in Fig. 4 and results in *I*_0_ = 85.67 μA cm^−2^, without taking into account transmission or reflection losses.

**Fig. 5:**
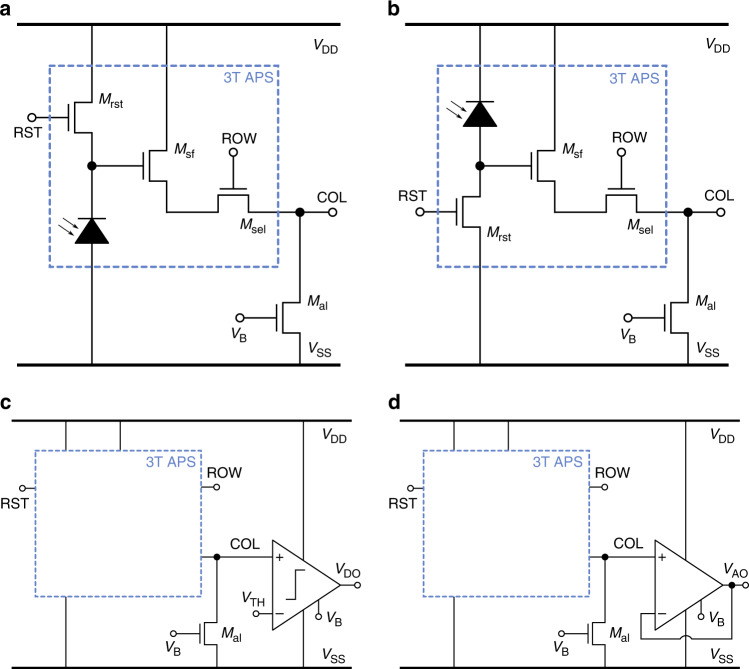
Pixel circuit design. Illustration of **a** the text book 3T APS in combination of the PW photodetector and **b** the alternative 3T APS in combination of the NW or SB photodetector. Both implementations require an active load biased by *V*_*B*_ to complete the source follower in the pixel. The pixel is controlled by a reset RST, row selection ROW, column output COL, high voltage *V*_*DD*_, and low voltage *V*_*SS*_. The design incorporates NMOS devices of 20 μm × 6 μm inside the pixel and two 20 μm × 10 μm in series as the active load. Both pixel types can be integrated with a **c** comparator or **d** unitiy-gain buffer for signal conditioning.

To design a scalable array of photodetectors, three transistor active pixels (3T APS) are employed which can be individually addressed and read out. The textbook 3T APS is illustrated in Fig. 5a and operates by storing charge in a MOS device, which then leaks away at different rates depending on the generated photocurrent. The initial charge is stored in the gate of *M*_*sf*_, which acts as a voltage source follower, using a reset pulse RST on *M*_*rst*_. The source follower output is connected to the pixel output COL through pass gate *M*_*sel*_, which is controlled by ROW. As the names suggest, COL and ROW are typically connected to the column or row of pixels in the array respectively. Finally, an active load is required to complete the source follower, which can be shared by the whole column. However, since the NW is implemented directly in the substrate, a conductive path exists between every n-well in the design. Since the body contact of the PMOS devices is connected to *V*_*DD*_ and implemented in the NW, so are the cathode connections of the NW and SB photodetectors. This implies that the textbook 3T APS is only possible with the PW photodetector architecture. A different 3T APS is illustrated in Fig. 5b which switches the roles of *M*_*rst*_ and the photodetector, meaning that the photodetector charges the gate of the source follower *M*_*sf*_ and *M*_*rst*_ discharges it. This architecture can then be used for the NW and SB photodetectors.

The two-pixel architectures are furthermore integrated with two CMOS circuit blocks for signal conditioning before further on-chip manipulation and off-chip interfacing. First, a comparator is added (see Fig. 5c) to effectively digitize and invert the photodetector response. Second, a unity-gain buffer is included (see Fig. 5d) to provide a buffered analog version of the photodetector response. Note that the integration of these CMOS readout blocks is indifferent to the choice of 3T APS topology.

**Fig. 6:**
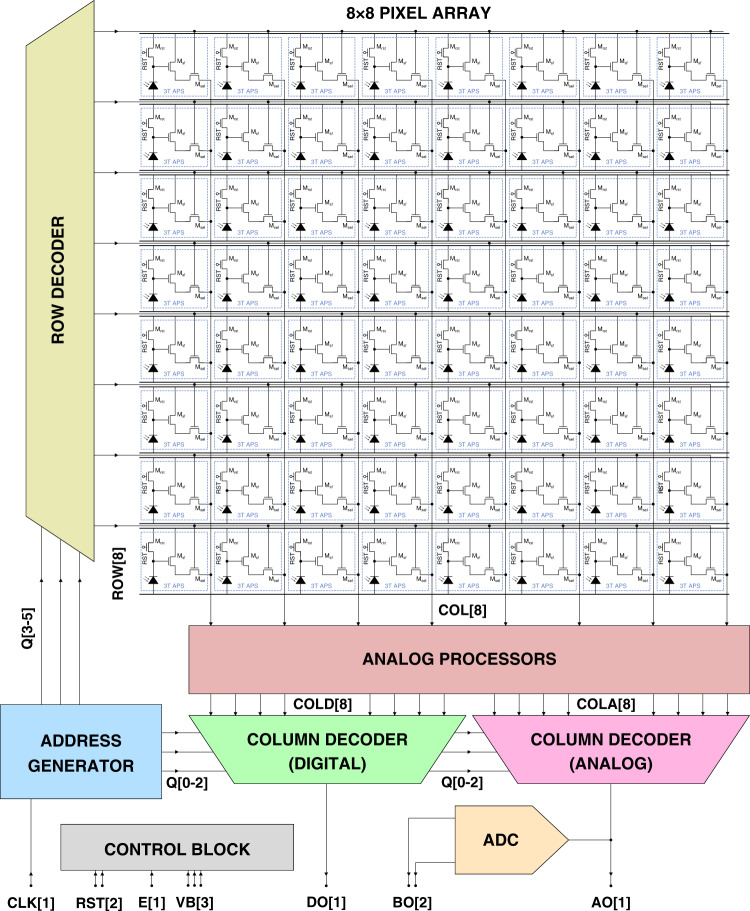
Functional block design. Schematic overview of the image array sensor system functional block design. The system is controlled by the global inputs CLK, RST, E, and VB and has three output channels DO, AO, and BO. Note that not all intermediate signals are depicted in favor of readability.

The image sensor houses an 8 × 8 pixel array and is integrated with control and readout electronics to implement a sequential system. The schematic block diagram is depicted in Fig. 6, of which the functional blocks are discussed in the following paragraph. The address generator sequentially iterates through all pixel addresses on a clock signal using a 6-bit address word Q. The most significant 3-bit half is fed to the row decoder and the least significant 3-bit half to the digital and analog column decoders. Each column COL in the array is connected to a channel of the analog processor, which passes the analog pixel values COLA as well as a digitized representation COLD. These two separate signal paths are connected to dedicated column decoders, which in turn pass a single digital DO or analog AO output off-chip. This implies that both DO and AO are 64-bit (8-byte) serial output streams. The analog output is furthermore connected to an ADC, which outputs a 2-bit representation BO of the analog output in parallel. Finally, the control block toggles on signal E between 7-pin or 10-pin control mode. The 7-pin mode allows for rapid wafer-level characterization to identify broken devices before packaging (the probe station allows up to 7 probe needles). The 10-pin mode provides control over separate bias levels and resets signals if required.

**Fig. 7:**
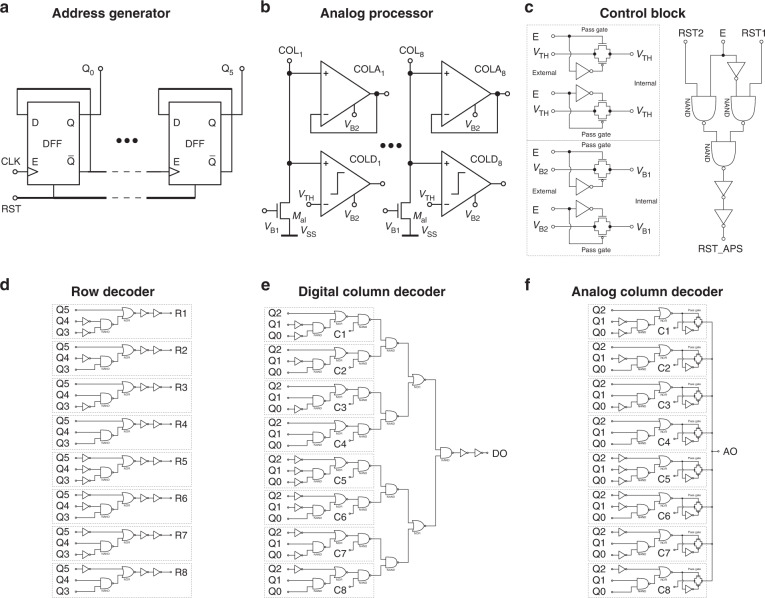
Circuit blocks. Circuit designs of the functional blocks in the opto-electronic system, including the **a** address generator, **b** analog processor, **c** control block, **d** row decoder, **e** digital column decoder and **f** analog column decoder. The used unit blocks are characterized in previous reports^23^.

The address generator (Fig. 7a) is based on d-flipflops to implement a 6-bit asynchronous binary counter. The analog processor (Fig. 7b) has eight channels that each consist of the active load (transistor *M*_*al*_), a comparator, and a unity-gain buffer. The control block (Fig. 7c) consists of a single digital 1-bit multiplexer and two analog 1-bit multiplexers. For E = HIGH, the internal signals are connected to their external counterparts (10-pin mode), but for E = LOW the internal analog signals are all connected to *V*_B2_ and the internal digital signals to RST1 (7-pin mode). The row decoder (Fig. 7d) is built from a NAND and NOR gate with two inverters to drive the ROW address lines. The digital column decoder (Fig. 7e) is implemented by a 3-bit multiplexer and the analog column decoder (Fig. 7f) is implemented by a comparable 3-bit multiplexer, with the addition of pass-gates that selected the analog signal.

## Results and discussion

### Discrete photodetectors

**Fig. 8:**
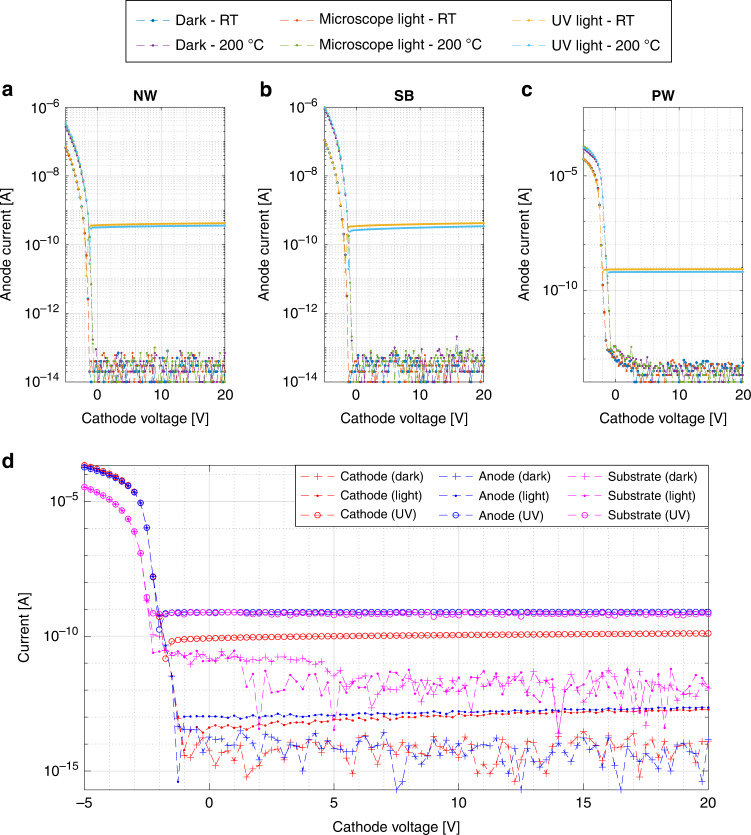
Photodetector IV response. The measured anode current for different cathode biasing and different temperature levels on die (−1,0) of the different vertical photodiode 80 μm × 80 μm implementations in **a** n-well (NW), **b** substrate (SB) and **c** p-well (PW). All signals are reported of **d** PW implementation for further analysis. The anode is biased at 0 V and the PW substrate connection at 20 V.

The photodiode response is measured using a MicroTech Cascade probe station with heated chuck that is paired to an Agilent 4156C Precision Semiconductor Parameter Analyzer and IC-CAP measurement software. The response of the three designs in Fig. 3 with a 50 nm thermally grown SiO_2_ active area is reported in Fig. 8a–c for different light conditions, bias, and temperature levels, showing strong responses to UV light that is constant over positive cathode bias. Since the NW and SB cathodes are connected to the substrate, the measured current at the cathode is generated at multiple devices and therefore not reported in the plot. In contrast, the PW implementation separates the cathode and substrate contacts and all signals are reported separately in Fig. 8d. As example, the PW80 UV photocurrent generation is 41 μA cm^−2^, which complies well with the predicted *I*_0_.

At a temperature of 200 °C, the most prominent observation is the unchanged dark current and lack of current generation under microscope light. Both the noise level and insensitivity towards visible light are thus unchanged in an environment of 200 °C, which indicates great potential for future applications that target higher temperature operation. Two minor changes in the IV curves are observed for the higher temperature level, which are a noticeable reduction of the generated photocurrent under UV exposure and a shift of the photodiode forward voltage towards lower bias. The photo-to-dark current ratio (PDCR) figure of merit^30,31^ is calculated by

2 $${{{\rm{PDCR}}}}(\lambda )=\frac{{I}_{photo}(\lambda )-{I}_{dark}}{{I}_{dark}},$$

where *λ* is the wavelength or bandwidth, *I*_*photo*_ the wavelength dependent generated photo current, and *I*_*dark*_ the dark current. The dark current and PCDR towards UV light are listed in Table 3.

**Table 3:** Overview of the PDCR results on wafer-level on 35 dies for different areas (labels refer to *L* in Fig. 3) and type of photodetector considering the anode current. For each respective case, the average anode current for a cathode voltage > 0 V is considered. The reported PDCR is proportional with the respective photodetector area.

Device	Dark current [fA]	PDCR (UV)
SB50	11.9 ± 1.6	(1.1 ± 0.3) × 10^4^
SB60	11.4 ± 1.0	(3.1 ± 0.7) × 10^4^
SB70	10.8 ± 0.7	(6.3 ± 1.4) × 10^4^
SB80	11.3 ± 1.8	(9.4 ± 3.3) × 10^4^
NW50	11.3 ± 0.8	(1.3 ± 0.4) × 10^4^
NW60	11.4 ± 1.1	(3.0 ± 0.8) × 10^4^
NW70	11.1 ± 1.1	(6.7 ± 1.6) × 10^4^
NW80	11.8 ± 1.6	(9.4 ± 3.0) × 10^4^
PW50	7.4 ± 0.8	(9.5 ± 2.1) × 10^4^
PW60	8.6 ± 4.0	(13.6 ± 4.1) × 10^4^
PW70	7.9 ± 0.8	(23.1 ± 4.8) × 10^4^
PW80	8.0 ± 0.7	(32.8 ± 8.3) × 10^4^

The devices show a very low dark current that is constant over the different device areas, resulting in averages of 11 fA (NW), 11 fA (SB), and 8 fA (PW). The dark current of PN photodiodes increases as the intrinsic carrier density increases, which is many orders of magnitude lower for silicon carbide, compared to silicon^32^. As a result, the typical dark currents are around 1 pA cm^−2^ and 1 fA cm^−2^ for silicon and 4H-SiC respectively^1,33^. Since the reported values are constant over area, the measured dark current is dominated by shared geometries such as the substrate and contacts. The NW and SB are therefore identical and differ from the PW, as the p-well is electrically insulated from the substrate and the contact silicides are different. This is also the reason for the unchanged dark currents at higher temperature, as the geometry-dominated noise sources are still larger.

For this reason, the actual PN junction dark current can not be measured. Normalizing the lowest reported dark current in Table 3 over the largest device area indicates that the actual PN junction dark current is <125 pA cm^−2^, which complies well with literature and shows that there is still room for improvement of the reported devices. The response to microscope light is very low, though noticeably higher for the PW implementation. This indicates a potential shift between the responsivity curves of the SB, NW, and PW implementations, which is not investigated further in this work due to the marginal difference. The PDCR towards UV light is above 1 × 10^5^ for the PW and above 1 × 10^4^ for the NW and SB implementations, making the PW implementation superior when measured at the anode. However, considering all PW connections in Fig. 8d reveals that the anode current is equal to the cathode current in the dark/light cases, but equal to the sum of the substrate and cathode currents in the UV case. This shows that generated photocurrent is leaking away to the substrate, reducing the signal to what is measured at the cathode about ten times. Future implementations should improve the PW architecture in Fig. 3 by suppressing the unintentional horizontal PN photodiode from anode to substrate, possibly by extending the PW.

**Fig. 9:**
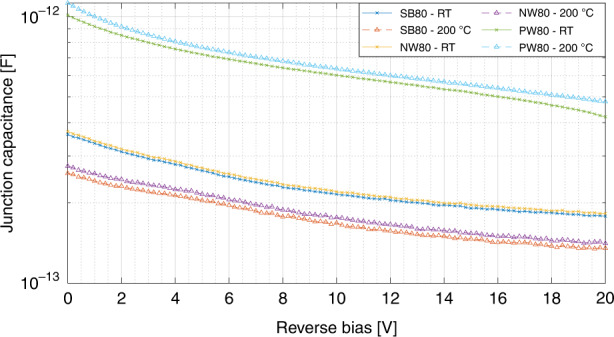
Junction capacitance. The measured junction capacitance at different biases and temperatures of the SB80, NW80, and PW80 photodiodes, using a 4284A Precision LCR meter at a frequency of 1 MHz.The capacitance of the SB and NW type decreases with temperature and that of the PW increases with temperature.

Next, the junction capacitance of the three photodetector types is measured over the complete bias range for different temperatures and the results are given in Fig. 9. As expected, the junction capacitance decreases for increasing reverse bias level, but remain in the same order of magnitudes (hundreds of fF). The NW and SB type behave similarly, while the PW junction capacitance is about three times higher. This is explained by the different doping concentrations of the NW and PW regions, which are 1 × 10^16^ cm^−3^ and 1 × 10^17^ cm^−3^ respectively, and the fact that the junction capacitance scales with the square root of the doping concentration. Note that the effect of temperature is opposite for devices in an NW or PW, which is ascribed to the opposite charge carriers of the device.

**Fig. 10:**
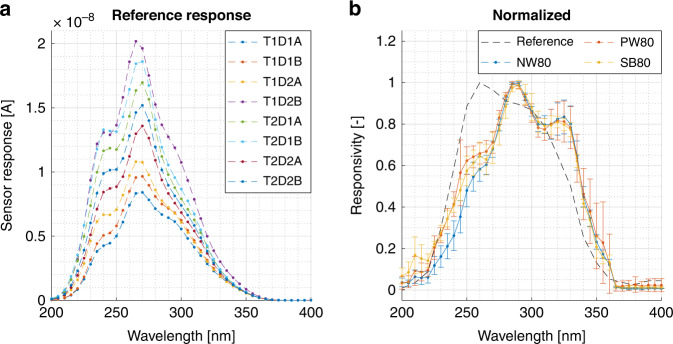
Photodetector responsivity. Sensor response of **a** eight identical reference devices, showing significant deviations in the magnitude of the response, and the normalized responsivity measurements of **b** the NW80, PW80, and SB80 devices. The normalization is done on the results of four different devices of the same type and then averaged to find the reported curves. Note that the standard deviation is derived as the deviation between the four normalized measurements results of each device type.

The SiC photodiodes are measured by an in-house UV responsivity measurement system, which employs an ILD-D2-QH Deuterium-Halogen UV light source from Bentham that is coupled to an iHR320 monochromator from Horiba to selectively cycle through the spectral range of 200–400 nm. A total of eight identical reference photodiodes^26,27^ were measured, indicating that the device response varies substantially in magnitude (Fig. 10a). Therefore, the measured responsivity curves of the three photodetector types are normalized for spectral comparison and the results are reported in Fig. 10b. The three photodetector types exhibit similar responsivity curves, which is attributed to the identical dopant depth profiles. Compared to the reference, the three devices types have a shifted peak responsivity from 260 nm to 290 nm. This shift is explained by minor differences in the doping profile or used ARC coating of the SiC CMOS design layers and the process used for the reference diodes, as this strongly effects the absorption depth for photons at these wavelengths^26^.

**Fig. 11:**
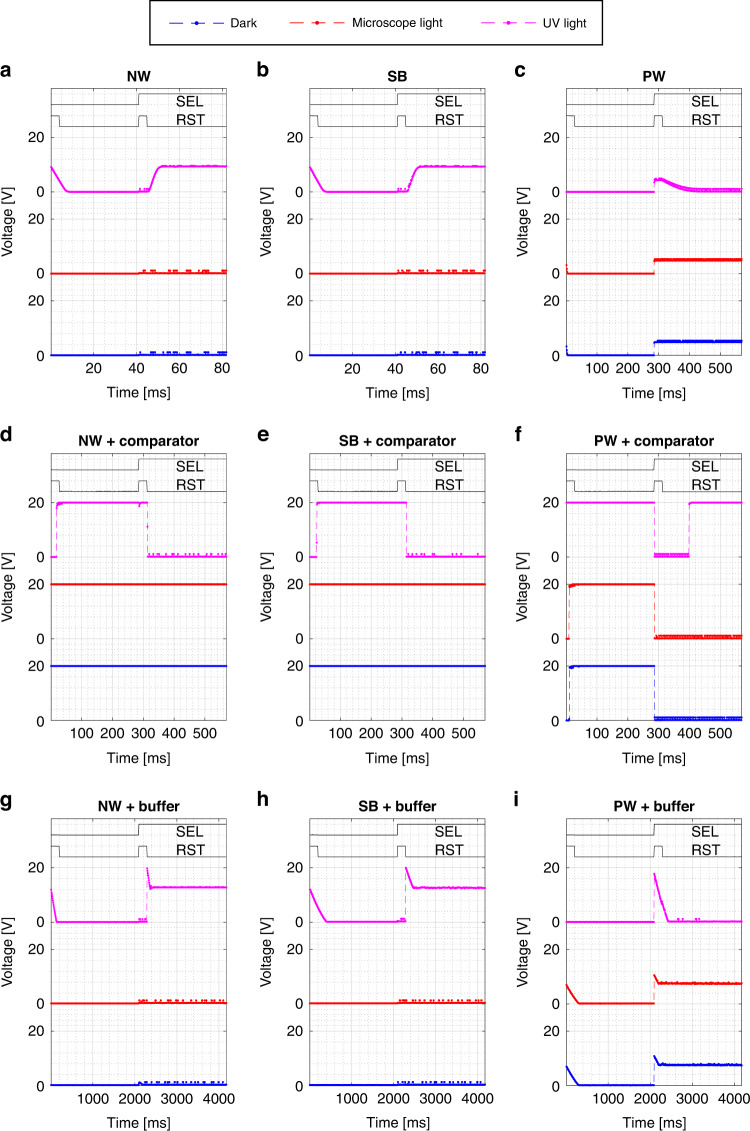
Active pixel response. The measured active pixel response on die (−1,0) of the different vertical photodiode implementations, including the **a**–**c** raw pixel output, **d**–**f** paired with comparator, and **g**–**i** paired with voltage buffer. The Fraunhofer IISB SiC CMOS supply voltage is 20 V and the active load of the raw pixel is biased at 6.0 V and when paired to a comparator or voltage buffer at 1.5 V. Each measurement is a segment of a continuous measurement to avoid start-up discrepancies.

The 3T APS are implemented by 0.25 mm^2^ photodetectors with a 400 nm LPCVD TEOS deposited SiO_2_ passivation layer and the circuits of Fig. 5. The time-dependent measurements are performed using a custom data acquisition tool that is controlled using a MATLAB GUI on a laptop. The data acquisition tool consists of analog 5-to-20 V and 20-to-5 V level shifters and an ATMEGA2560 microcontroller that is loaded with the measurement code. The raw pixel output results are given in Fig. 11a–c and the illumination conditions in Fig. 8 are considered when the pixel is disconnected (SEL = LOW) from and connected (SEL = HIGH) to the output. As expected from the single photodetector results, the NW and SB perform near identical and charge to a *V*_*high*_ of (9.4 ± 0.2) V under UV illumination, while remaining at low voltage in the other cases. The PW implementation is reset to a *V*_*high*_ of (5.0 ± 0.3) V and discharges to low voltage under UV illumination, while remaining at high voltage in the other cases. The NW and SB charge time is (7 ± 1) ms and the discharge time of the PW is (87 ± 7) ms, which corresponds closely to the reduced PW signal of about ten times due to the unintentional PN junction to the substrate.

The pixel response times are high and will therefore negatively impact the sensor readout speed. The found response times are verified by considering the reported photocurrent generation (Fig. 8) and junction capacitance (Fig. 9), scaled for the larger photodiode area in the pixel. This implies that in order to reduce the pixel response times, research efforts should focus on increasing the photodetector quantum efficiency and reducing the junction capacitance.

Two CMOS circuit blocks are connected at the APS output for signal conditioning. First, a comparator^23^ is integrated to effectively digitize and invert its response and the results are listed in Fig. 11d–f. The NW and SB response time is near identical to that of the raw pixel, while the PW response time is about 7 ms longer. Second, a unity-gain buffer^23^ is integrated at the APS output, replacing the comparator, and the results are listed in Fig. 11g–i. The reset pulse brings the buffer output voltage to a high level in the UV case for all three architectures. The NW and SB response times are (151 ± 67) ms and (168 ± 50) ms respectively and the PW decay time is (378 ± 160) ms. The voltage buffer is not able to keep up with the APS signals for the applied UV intensity, which therefore only allows for application for steady state readout or much lower UV intensity situations.

### Integrated readout electronics

**Fig. 12:**
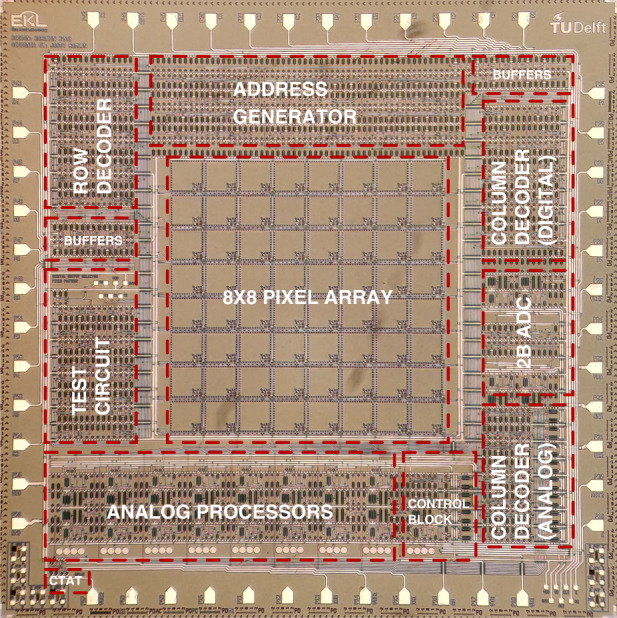
Image sensor system. Micro photograph of the 64 pixel array sensor with integrated readout electronics in 4H-SiC. The functional building blocks are highlighted and the incorporated pixels are the 3T APS design in Fig. 5a with the PW photodiode architecture. The optoelectronic system is implemented on a 10 mm × 10 mm chip and can be read out using only 7 pins, with many more intermediate signals available for validation of the design.

The conventional 3T APS is used in the 64 pixel array with integrated readout circuits, of which the fabricated chip is highlighted in the microphotograph in Fig. 12. All signals passing off-chip go through buffers that are capable of driving larger loads. The image sensor system is integrated with a previously reported on-chip five transistor diode-based CTAT sensor^34^, enabling the system to monitor temperature in future applications. Before the image sensor is measured, it is vital to investigate some of its functional blocks.

**Fig. 13:**
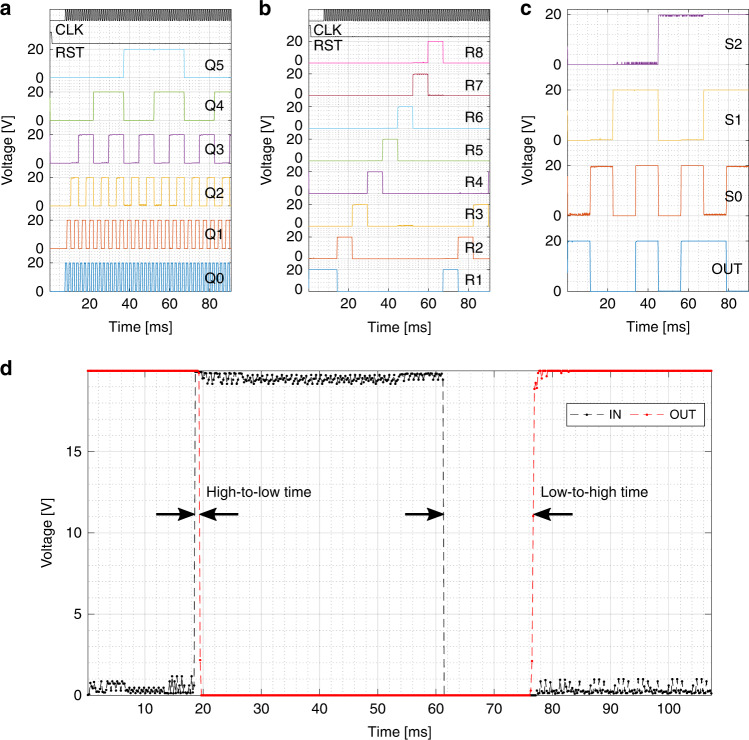
Functional block verification. The measured response of the **a** 6-bit address generator, **b** row decoder, **c** fixed pattern output selector on die (−2,−1) and **d** comparator on die (−1,0). The 6-pin fixed pattern output selector test structure and comparator were measured on wafer level, while the 10-pin address generator and 12-pin row decoder could only be measured on chip level after packaging due to the increased amount of pins.

First, the response of the address word generator is given in Fig. 13a, which is implemented by a 6-bit counter and performs as designed as its LSB flips state for every rising edge of the CLK input. Next, the row decoder response is provided in Fig. 13b, which takes the three MSB of the address (Q3–5) as its input. The row decoder operates as designed by iterating through all eight rows, while never selecting two rows at the same time. Finally, the digital output selector is investigated by the implementation of a separate test structure with a fixed output pattern. This fixed pattern is formed by connecting the column inputs to either *V*_*DD*_ or ground and is set to ‘10010110’. The digital output selector takes the three LSB of the address (Q0–2) as its input, which are here provided from an off-chip source as S0–2. The measured response is given in Fig. 13c and shows the correct output. The used clock frequency in these measurements is 1 kHz with eight data points per clock cycle. The response times for all these blocks are below the time step resolution of the measurement, from which it is concluded that the digital control and readout circuits are capable of operation at frequencies above 8 kHz.

The comparator^23^ response times are characterized and a typical result is given in Fig. 13d. It was found that the high-to-low transition time is (1.4 ± 0.4) ms and the low-to-high transition time (24.6 ± 13.1) ms, with outliers of 52 ms. This asymmetry is undesirable and becomes addressable when the available design models are no longer limited to DC analysis only, which is the case presently. If considering 100 ms to ensure all comparator outliers are caught, the analog processor blocks are limited by a maximum operating frequency of 10 Hz and are thus the rate-limiting block when considering the complete image sensor.

### Image sensor

**Fig. 14:**
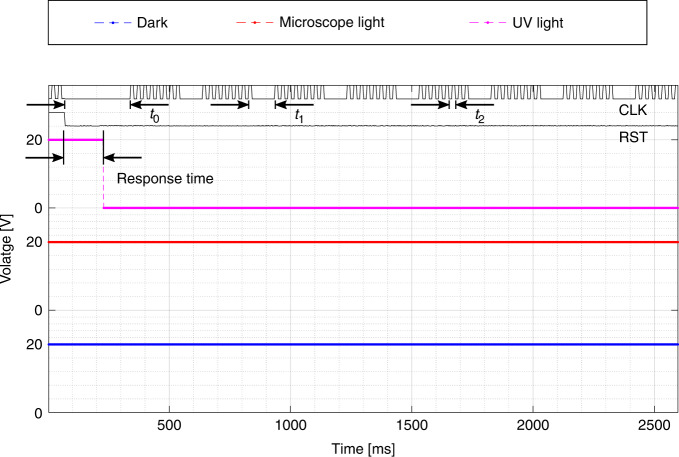
System timing scheme. The measured response to different illumination cases of the **a** pixel array sensor on wafer level on die ( − 2, − 1), by connecting the input *E* of the control block in Fig. 13 to ground and setting the bias to 1.5 V. The control signals reset ‘RST’ and clock ‘CLK’ are annotated, as well as the clock timings *t*_0_, *t*_1_, and *t*_2_.

The image sensor is read out on wafer-level using its 7-pin mode and the response for different illumination modes is given in Fig. 14. The image array consists of 64 PW 3T APS photodetectors, that have a fill factor of 77%. There are three different timings used in the clock signal, which are response times *t*_0_ for the individual pixel, *t*_1_ for the comparator, and *t*_2_ for the digital circuitry. When considering a continuous clock signal, the longest of these response times must be taken as the time of one clock cycle. This is *t*_0_ and is set to 274 ms, which would result in a total time of 35 s, or 29 mHz, for the full array. Instead, an asynchronous clock signal is applied that is tailored for each respective response time, to reduce the total readout time.

First, the system is reset for two clock pulses. Second, all pixels need to respond to the illumination and time *t*_0_ is waited before switching from the first to the second pixel. Thirdly, each time that the system moves to the next row in the array, all the column analog processors need to respond. For the digital readout, this concerns the comparator and a *t*_1_ of 108 ms is waited before switching from the first to the second column in the row of pixels. Finally, the digital output selector responds to moving through the columns in the array. For this, a *t*_2_ of 27 ms is waited between pixel switches. The clock timings are conservative considering the reported response times in the previous section, to ensure that any potential deviations are covered. This brings the total readout time down to 2.6 s, or 0.39 Hz, which is a 13 times improvement to the synchronous clock. As a fair comparison to the reported 256 pixel array in a SiC BJT technology^15^ the timing scheme is extrapolated, resulting in a total time for 256 pixels of 8.4 s, which is an improvement of 15 times.

**Fig. 15:**
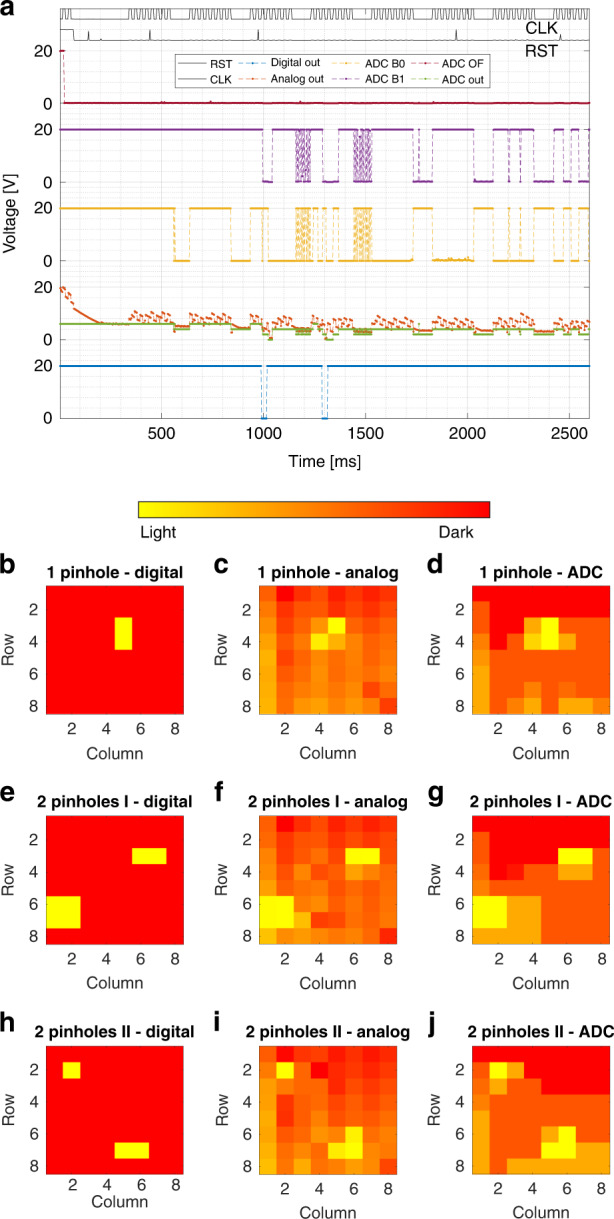
Captured images. Sensor outputs on die (−2,−1) for UV illumination through a paper mask. The serial outputs include **a** digital output, analog output, and 2-bit ADC output. The serial data is conditioned and transformed to 2D images for the cases of **b**–**d** one pinhole, **e**–**g** two diagonal pinholes, and **h**–**j** two diagonal pinholes rotated by 90°. The column readout direction is reversed for the digital and analog output selectors, which is corrected in the 2D images. The control block input *E* is connected to ground, the bias is set to 1.5 V and the ADC range is set to 8 V using external voltage sources. The spikes in the RST input signal are artifacts of the measurement tool and do not affect the sensor.

The image sensor is read out on chip-level using the 7-pin mode set by the control block (see Fig. 12), as this proved adequate for correct operation. Furthermore, the other two image sensor output modes are now also measured, which include the analog output and 3-pin ADC outputs. Since only dark and light measurements do not verify if an actual image can be made, simple paper masks with one or two pinholes were put on the chip. The raw sensor outputs for a mask with a single pinhole are listed in Fig. 15a, where the ADC out value is calculated using the 2-bit output from the ADC and plotted over the analog curve for ease of comparison. It was found that, due to a minor design error, the readout direction of the columns is opposite for the digital output selector compared to the analog output selector, which means that the clocked outputs in Fig. 15a of the digital and analog readout cannot be directly compared. Instead, the resulting image found for the digital readout is flipped over the columns before presenting in Fig. 15. The digital output shows that two adjacent pixels are illuminated through the pinhole, without any other pixels responding falsely. The interpretation of the serial analog outputs is less clear, as the output level jumps from pixel to pixel and is still settling during each pixel readout. Still, one can find two dips in which the analog outputs fall close to zero. The ADC output again clearly indicates two moments where the pixel output goes to zero, while maintaining a much more constant value during pixel readouts. Another observation is that both the analog and ADC output settle to lower levels during the longer readout times when moving to the next row in the array. This is likely due to switching effects in the circuit, as all column analog processors are connected to new pixels when moving to the next row.

The raw sensor outputs for different masks are conditioned by transforming the serial data stream to a 2D image, of which the results are listed in Fig. 15b–j. The sensor outputs are sampled eight times per clock cycle, which allows for averaging over eight data points to determine each pixel value. When considering all eight output data points sampled during a clock cycle, it was found that the first pixel in the row (column 1) always had a lower analog value, which is due to switching effects from moving to the next row. This effect is greatly reduced by considering only the second half of each clock cycle of four data points. The 2D images all verify the image sensor operation as the pinholes are clearly visible.

The power consumption of the image sensor is determined by measurement of the DC input current at the positive power supply terminal using an HP 34401A multimeter. Without UV illumination, the power consumption is 8 μW and 30 μW when idle and during readout, respectively. This is increased for UV illumination to a power consumption of 30 μW and 60 μW when idle and during readout. It should be noted that in the case of no UV illumination, after switching on the power supply and before a first readout is started, the input current is large (0.13 mA) and decreases in minutes to the corresponding idle value. Starting a sensor readout immediately brings the power consumption down to the reported value. As such, it is recommended to perform a power-on reset for the system. The reported power consumption of the 256 pixel array in a BJT technology^15^ is 8.25 W, which implies that the sensor reported in this work has a reduced power consumption by a factor of 137,500, considering its maximum measured power consumption.

The reported CMOS UV optoelectronic system in SiC is implemented on a 10 mm × 10 mm chip and includes 1236 transistors, which allows for digital, analog, and discretized outputs to capture 8 × 8 images in the UV range. In comparison, the first commercially produced microprocessor, Intel’s 4004 in 1971, includes 2300 transistors. This opens the door toward higher complexity systems in SiC for UV optoelectronics, as well as different applications that exploit the potential of high-temperature operation.

## Conclusion

An integrated 64 pixel image sensor in a 6 μm 4H-SiC CMOS technology is presented. Three photodetector architectures, that use the NW, SB, or PW design layers, are investigated individually and with integrated CMOS readout blocks connected to them. The measured PW PDCR to UV is largest, but due to an extra unintentional PN junction in the PW design, the NW and SB currently have 10 times faster response times. The digital circuit blocks in the sensor functional blocks are verified and found able to operate at above 8 kHz. The image sensor incorporates the PW photodetectors and offers a digital, analog, and 2-bit ADC output that are all verified of correct operation by light/dark measurements and illumination through a pinhole mask. The device has a refresh rate set to 0.39 Hz and a maximum power consumption of 60 μW, which are both improvements over previous reports of image arrays in silicon carbide.

The individual photodetectors are investigated at 200 °C and show unchanged dark current with negligible degradation of the PDCR towards the UV light source. Similarly, the effect of temperature on the photodiode junction capacitance is minor, from which similar behavior can thus be expected for the active pixels and therefore the image sensor system. Further investigation would benefit from packaging solutions that allow the direct measurement of the image sensor in high-temperature environments.

Future work on optoelectronics in SiC CMOS would benefit from improvements in fabrication technology, circuit modeling, and the presented hardware design. It was found that the dark current of each architecture is dominated by other factors than the PN device and benefits from improved ohmic contacts and device isolation. The most promising PW architecture is further improved by optimizing the layout design to avoid significant leakage current to the substrate. The addition of antireflective coatings in future implementations will contribute to further enhancement of the photodetector response. Circuit blocks such as the unity gain voltage buffer and comparator are optimized by extending the modeling capabilities beyond DC analysis. Assuming the faster NW and SB photodetectors, symmetrical comparator response times, and reported response time of the digital circuits, the device refresh rate is predicted to exceed 10 Hz in future implementations.
